# How the Language of Instruction Influences Mathematical Thinking Development in the First Years of Bilingual Schoolers

**DOI:** 10.3389/fpsyg.2021.533141

**Published:** 2021-04-13

**Authors:** Vicente Bermejo, Pilar Ester, Isabel Morales

**Affiliations:** ^1^Evolutionary Psychology, Universidad Complutense de Madrid, Madrid, Spain; ^2^Facultad de Educación, Universidad Camilo José Cela, Madrid, Spain

**Keywords:** bilingual programs, mathematical thinking development, language of instruction, mother tongue, content and language integrated learning

## Abstract

The present research study focuses on how the language of instruction has an impact on the mathematical thinking development as a consequence of using a language of instruction different from the students’ mother tongue. In CLIL (*Content and Language Integrated Learning*) academic content and a foreign language are leant at the same time, a methodology that is widely used in the schools in the present times. It is, therefore, our main aim to study if the language of instruction in second language immersion programs influences the development of the first formal mathematical concepts. More specifically, if the learning of mathematical concepts in the early ages develops in a similar way if it is taught in the students’ mother tongue and is not influenced by the language used for teaching. Or else, if it can influence the development of the first skills only in the students’ general performance or in certain areas. The results of both the analysis of variance and multiple regression confirm how influencing the language of instruction is when mathematical thinking is developed teaching formal contents in a non-coincidence language. The second language is affecting the resolution of daily life problems, being more competent those students in 1st grades whose language of instruction matched with their mother tongue.

## Introduction

The importance of learning and becoming proficient in an additional language has become a milestone in educational policies, and subsequently in the pedagogical lines of many educational institutions in recent years. Consequently, English as the language for international scientific-technical communication keeps away from the rest of the languages of the European Community, becoming the core of bilingual policies based on the so-called CLIL educational approach.

Despite the criticism that bilingual programs have gone through and are still currently suffering, the students of such programs are demonstrating how beneficial it is to obtain very good results in the university entrance exams (Fiedu and Haro, 2017).

On many occasions, mathematics has not been considered a suitable subject to be taught in a language other than the mother tongue, a decision made sometimes because of the complexity and abstraction that this subject possesses in itself. However, there are international schools that implement immersion programs where the second language is acquired naturally and tend to teach the entire curriculum in that language, with the exception of Spanish language instruction.

Therefore, we find it interesting to study if subjects such as mathematics, and more specifically, the learning of mathematical concepts in the early years develops in a similar way regardless of the language of instruction, or else if it can influence the development of the first skills only in general performance or in certain areas. In other words, does the language of instruction influence the development of the first formal mathematical concepts?

In order to find an answer to this question, the present work analyzes various mathematical tasks such as counting, number line, resolution of algorithms and verbal problems in students who are studying in first and second grades of elementary education in different international schools of the same educational institution. In each grade, one group of students is taught mathematics in Spanish and another group in English. We have analyzed the execution of activities in two differentiated groups: the group whose mother tongue coincides with the language of instruction and the group whose mother tongue does not coincide with the language of instruction.

## Teaching in Bilingual Contexts

It is unquestionable that there exists a relationship between mathematics and language. But how, when and in which tasks is language more straightforwardly influential?

To answer this question, it is necessary to delve into the different levels of language acquisition, and not only to rely on the superficial advantages or disadvantages that bilingual teaching offers, but also assessing to what extent the development of mathematical thinking can be constrained if it is taught in an additional language or a ‘non-dominant language.’

Therefore, it is essential to highlight the existing difference between Second Language Learning (SLL) and (SLA) Second Language Acquisition and the way these concepts directly influence the action of facing new content and learning naturally. Acquisition as a natural and unconscious approach is close to the phenomenon that take place when the first language is acquired. We can identify a consecutive bilingualism, either due to a linguistic immersion through an educational program, or as a product of what is known as submersion in a communicative context where the child lives with native speakers (Snow, 1999). Concerning language learning, we should not fall for the instruction of a L2 as it was carried out in many educational environments applying traditional methodologies, focusing on learning vocabulary and reading comprehension.

It is also relevant to remember and emphasize that not all bilinguals have become bilingual in the same way, and for that reason their degree or type of bilingualism is also different. We must then differentiate between ‘balanced bilingualism,’ that is, individuals using two languages on a regular basis, and ‘dominant bilingualism’ referring to those bilinguals who are more proficient in one language as compared to the other language. We resort to this taxonomy of bilinguals, because it is the one that fits better with the study and relates to language proficiency and competence development.

Concerning the age of acquisition, we can also distinguish three ways to acquire more than one language, *simultaneously*, when a child learns two languages from birth, or *sequentially*, when one language is acquired after another, and *receptively*, when bilinguals do not have opportunities to use the additional language but are likely to understand a great deal. According to Baker (2011): ‘…simultaneous childhood bilingualism refers to a child acquiring two languages at the same time from birth, sometimes called infant bilingualism, bilingual acquisition and bilingual first language acquisition’ (p. 94). In addition, the author points out that sequential acquisition refers to the situation when a child or adult acquires a first language and then acquires the second language or additional languages.

Since a foreign or second language is included in the Infant Education curriculum as a subject-matter like the rest of the contents, we also found necessary for our study to take into account the differences between bilingual education and he application of CLIL methodology. According to Bentley (2010), it consists in applying a form of partial immersion where half of the curriculum or more is taught in the non-native language known as ‘hard CLIL.’ Or else, ‘soft CLIL’, it is a methodology more suitable for early ages, since second language teachers work or teach any curricular content as part of language teaching in a more holistic way (Ball, 2009). This will allow learners to explore any content from a different perspective while they are also improving the foreign language. For instance, teachers can work on transport in history and mathematics, carry out multidisciplinary and global projects, and reconceptualize the curriculum in an integrated way (Garciìa, 2015).

The implementation of CLIL approach in ‘partial immersion’ is increasingly widespread in schools, where Spanish language and mathematics are taught in Spanish as the first language. However, a ‘total immersion’ in an additional language is the methodology that is causing many schools to teach mathematics in that language. As it is the case in the present study.

A fact of special interest for our research is related to the results obtained by students in other subjects within the immersion programs (Sotoca, 2014). These are not affected by the fact that these subjects are taught in a second language. And, if there is any delay in reading, writing and mathematics in immersion students, it disappears later at sixth grade (Turnbull et al., 2003).

## Mathematics and Language

Various studies on how the linguistic structure of a problem can favor or block the resolution of the problem by the students makes clear the extraordinary relationship between language and resolution of verbal problems (Carpenter and Moser, 1984; Verschaffel et al., 2000; Bermejo et al., 2002, 2021). It is also worth mentioning the advantages of the cognitive function in bilingual children compared to monolinguals. Ellen Bialystok (2009) showed how bilingual children between 4 and 8 years old have great advantages over monolinguals when solving problems because they controlled their attention; they were not distracted by more confusing and misleading aspects, and they even better discerned the appearing reality and by demonstrating an improved cognitive function. Bialystok (2018) suggests that the bilingual experience leads to an adaptation of the central executive component of Baddeley (1986) WM (Working Memory) model even in young children. However, authors such as Volmer et al. (2018) suggest that certain skills depend on the language through which they were developed and acquired at least partially, which does not mean that they always represent an advantage.

**FIGURE 1:**
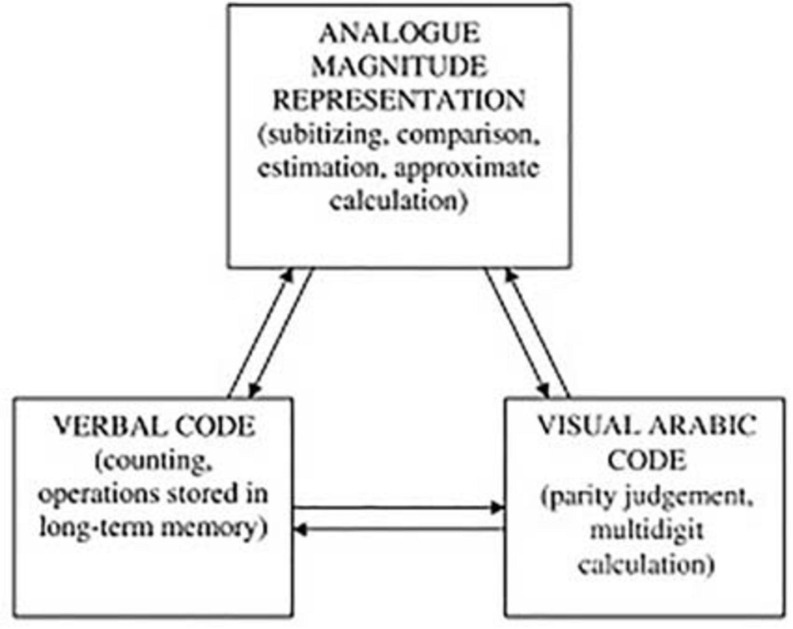
Dehaene triple code model (1992).

Nonetheless, when we talk about the development of mathematical thinking in the early ages, we refer to a great deal of constructions of basic concepts as well as the acquisition of mathematical procedures that allow us to develop problem solving, which is one of the most complex tasks (NCTM, 2000). Tasks such as counting, calculation, measurement approximation, comparison of magnitudes and problem solving are some of the mathematical contents that the child learns and develops throughout his childhood. Some authors (McCloskey, 1992; McCloskey and Macaruso, 1995) state that any number we perceive is recoded in an ‘amodal representation’ prior to performing its processing. This theory would rule out that some of the concepts developed have had a direct relationship with language. However, Dehaene (1992) and Dehaene and Cohen (1995) propose three types of internal representations that can be involved in solving tasks: an analog magnitude, an arabic visual code and a verbal system (see Figure 1). This leads them to conclude that there are great connections between the internal representations performed and the language in all the mathematical tasks that are carried out.

Campbell and Epp (2004) clarify that the numerical processing is carried out in different codes, therefore they are more inclined to use different codes, depending on the task, which would rule out a unique relationship with language in all tasks made.

First, the child develops a concrete or perceptual arithmetic that later becomes abstract through the use of oral language, and second, he develops the writing based on the representation of the number with the help of digits. This step from perceptual to abstract arithmetic is far from being scientifically described at the moment, but it is assumed that there exists an activation of different parts of the brain. What is still unclear at the moment is how this transition from concrete-perceptual to an abstract-symbolic arithmetic is realized and then concretized by digits. Some studies carried out through neuroimaging techniques show how working memory is highly important in complex calculation tasks, and indicate the way working memory uses visual or verbal procedures depending on the strategies that individuals use (Delazer et al., 2003).

Concerning calculus operations, Colomé et al. (2010) conducted a study in Italian monolinguals and Basque-Spanish bilinguals. These authors departed from the hypothesis in which, if the language influenced the calculation even when the presentation was in arabic numbers, the Basque-Spanish bilingual participants would have an advantage over the Italians because the linguistic structure of the numbers, focused on the base 10 and 20, matched with that of its dominant language (Basque). The results of these studies indicated that there were specific effects due to the language use. As the authors indicate: “it seems that the word that designates a number has an effect on the processing of a number” (p. 967).

In this sense, neuroimaging studies have shown how specific areas of language are activated when calculation tasks are performed, sustaining the existence of a language-dependent system as opposed to independent numerical systems (Pesenti et al., 2000; Benn et al., 2012). Therefore, it seems clear that quite a few authors propose different approaches to show the high influence of language on mathematical thinking. However, the studies carried out by Swanson et al. (2019) show how the influence of certain aspects such as reading and vocabulary does not have as much influence on problem solving as working memory does, where a great correlation between these results is obtained in bilingual children, in contrast with monolingual children, also shown in the studies by Swanson et al. (2018). Bernardo and Calleja (2005) also showed how the students’ linguistic competence in the language of instruction directly influenced the development of their own competences, specifically in mathematics. We find such an ability similar to that of counting.

Other studies show how German–French bilingual students could transfer their capabilities in approximate calculation tasks from one language to another, but they could not perform this transfer for exact calculations (Saalbach et al., 2013). However, Salillas and Wicha (2012) show that bilingual students of higher courses can perform calculations more effectively in the non-mother tongue, if this has been the language of instruction used in the classroom.

We should also consider the fact of how instruction is developed in a second language. Some studies show how direct instruction and training teachers through programs based on visualization and guided by the choices they can make during such an instruction, can improve teaching in the early ages and subsequently their students’ performance. Other studies such as those of Kraft and Hill (2018) reveal how teacher training allowed them to detect critical points to improve mathematics quality teaching to incorporate them into their day-to-day life; it substantially improved the quality of their classes and their students’ performance.

## The Present Study

One of the most relevant issues when conducting the research on bilingualism is how each of the students has acquired the second language, or if their learning has occurred simultaneously. Factors such as knowing the time of exposure to the language, the competence shown by the student and the informal experiences they have had, are determining factors that can contribute to make significant differences between them. Our study is conducted in two international schools where linguistic immersion is being carried out from the first years of schooling, that is, from the first years in nursery school, that is, since the age of 4 months. And, although some of the students are incorporated later, we must highlight that a large majority of the sample has been attending school at least since the age of three.

Conducting a study on the influence of the language of instruction in international schools is quite advantageous. On one hand, because a high number of students have had the same time of exposure to the second language, mostly with English native teachers, and on the other, because the teaching method in both groups is quite similar, as it follows the same IB methodology implemented in all international school centers. Furthermore, this research is of special interest because it is carried out in early year students during which the linguistic competence in both the mother tongue and the instructional language is not completely proficient in all students.

As above-mentioned the participants belong to first grades and second grades of elementary education in two international schools in Spain, both being taught through the International Baccalaureate (IB) philosophy and teaching methodology and instructed with the same linguistic immersion in the second language or language of instruction. Along the different years, students are taught 100% immersed in English. Accordingly, one of the schools, school *A* teaches all subjects of elementary grades in English, except for the Spanish language. And the school *B* that uses the Spanish language to teach both Spanish language and mathematics.

## Materials and Methods

### Participants

To carry out the present research, there was a total sample of 169 bilinguals studying in international schools. The sample was made up of 80 first grade students (39 girls, mean age of 7.1 years and 41 boys, mean age of 7.3 years); and 89 second grade students (38 girls, mean age 8.2 years, and 51 boys, mean age 8.2 years).

All participants had the same linguistic immersion in the second language. They spoke both Spanish and English. However, as it is an international school there are students from different countries, origins and nationalities: Brazil, United States, Portugal, England, and Italy. Each of these schools was characterized by the use of a different language of instruction in the subject-matter of mathematics. School *A* taught it in English whereas School *B* taught it in Spanish. For this reason, for the analysis we decided to form two differentiated groups, not by school, but by their mother tongue, that is, if their mother tongue matched or not with their language of instruction and how the students used it. Hence, we formed four different groups. Group 1 would be formed by first grade students whose mother tongue coincided with the language of instruction, and group 2 would be formed by first grade students whose mother tongue did not match with the language in which mathematics is taught. Group 3 would be made up of students whose mother tongue did not match with the language of instruction, and finally group 4 would be formed by students whose mother tongue matched with the language of instruction. In this way, the sample was as follows: out of the 80 students in first grade 44 students made up group 1 (24 girls and 20 boys), and 36 students made up group 2 (15 girls and 21 boys). The sample of second grade students is formed by 49 students in group 3 (20 girls and 29 boys) and 40 students in group 4 (18 girls and 22 boys).

### Materials

Students performed several mathematical tests to assess their mathematical proficiency and several tests to assess the homogeneity of the groups. In first grade groups, we used a chronometer to measure the time devoted to solving mathematical verbal problems and find out that there were time differences in their resolution, since one of the groups received instruction in the Spanish Language for the first time.

The tests that were applied were the following:

1. The *Raven CMP* test (Raven et al., 1996). This test measures general intelligence and the “g” factor. It is a collective application test that consists of a booklet with three series of matrices called A, Ab, and B. The series Ab discovers the student’s ability to establish relationships between isolated figures, and the series A and B cover the whole cognitive process of children up to 11 years of age. Our main purpose was to assess whether there were significant differences in this factor among participants. The reliability and validity of this test presents a reliability of 0.87–0.81, while in validity an index of 0.86 was obtained. These data were obtained with the Kuder–Richardson formulas and with the Terman Merrill criteria.
2. The *Difference Perception Test* (TPD) (Thurstone and Yela, 2012). It is based on the following measurements: (1) right (A) correct face crossed out; (2) errors (E) faces crossed out without being correct; (3) net (A − E), the part that measures the student’s real effectiveness once the errors have been penalized. It is calculated by subtracting the total number of errors from the total number of correct answers; and (4) the Impulsivity Control Index (ICI) that measures the cognitive style of the reflexive-impulsive subject and is calculated by using the following formula: ICI = *A* − *E*/*A* + *E*. The result shows the evaluation of the attentional processes in the participants. This instrument has an internal validity = α = 0.76, and the test-retest reliability of 17 out of the 20 items with a coefficient greater than 0.6, and 14 of them greater than 0.8 implying an excellent test-retest reliability.
3. *Tema-3* consists of several tests that are used to evaluate the mathematical proficiency. It is an individual test carried out with pencil and paper to assess formal and informal skills. These items are distributed according to age and divided into several aspects that value both informal (41 items) and formal concepts (31 items). The 41 items that assess the most informal aspects of mathematical thinking are divided into four large fields: (1) numbering (i.e., mastering the numerical sequence through counting, numbering, etc.); (2) comparison of quantities and establishing the distances between different numbers; (3) informal calculation, such as simple additions and subtractions, and mental calculation tests using manipulable materials; (4) basic concepts such as the application of superior counting strategies, distribution with concrete objects, the cardinality rule, etc. The formal part is evaluated by using 31 items that are divided into four large groups: (1) reading-writing of quantities; (2) strategy of numerical facts; (3) formal calculation; and (4) basic concepts related to the decimal number system. The application of this test provides us with individual information to assess the level of mathematical competence, as well as the percentile in which the student is ranked, but it is especially useful concerning the information it offers on the difficulties and potentialities per individual. (Test of Basic Mathematical Competence; Ginsburg and Baroody, 2003). The test is validated in the Spanish population with a reliability index (Cronbrach’s alpha = 0.92), a validity backed up as a measure of early mathematical competence (Ginsburg and Baroody, 2007).
4. *Tedi-Math* operation subtests complement those areas evaluated with *TEMA-3* tests. It is another standardized test that measures the mathematical competence. It is made up of 25 subtests grouped in different areas: Counting, numbering, understanding the number system, doing operations and solving verbal problems. We decided to use only the subtest of operations as an adequate complement of the areas evaluated with *TEMA-3* tests. *Tedi-Math*’s operation subtests consist of eighteen simple additions and four additions with gaps, two of them with the unknown in first place and two of them with the unknown in the second, without exceeding number ten. It also contains fourteen simple subtractions, four subtractions with gaps, two with the unknown in the minuend and two in the subtrahend, and similarly as in additions, none exceeds number 10. And the test was completed by giving fourteen simple multiplications. The Cronbach’s alpha corresponding to the test was (α = 0.93). For the subtest used, the reliability indices are as follows: arithmetic operations (α = 0.99) and size estimation (α = 0.95).
5. Twenty addition and subtraction verbal problems were also administered individually. None of the problems exceeded number twenty in the expected answer. The answer was recorded as correct when the student knew how to explain the result provided. When the answer was given at random, they were considered invalid. The problems were selected and extracted from the investigations carried out by Bermejo et al. (2002), and were sequenced in ascendent difficulty according to the classification carried out in the above-mentioned study: 8 change problems (four addition operations and four subtraction operations with the unknown in the beginning, middle and end of the equation); 6 comparison problems (three additions with the unknown in the referent and in the comparison, and three subtractions with the unknown in the referent and in the difference); 2 combination problems (one with the unknown in the beginning and one in the middle) and four equalization problems (two additions with the unknown in an unknown set, in the unknown equalization, and two subtractions with the unknown in the unknown equalization, in the known set). This subtest was conducted in the language of instruction used to teach the subject of mathematics: in Spanish to students who received mathematical instruction in Spanish, and in English to students who were taught in English. The translation of the problems was carried out following the established procedure (Muñiz et al., 2013).

### Procedure

Two collective tests (1 and 2) were given first to the groups in their reference classrooms during their school day. Then, the students were assessed individually according to the language of instruction in which mathematics was taught in their school during the day. It took place in a room close to their reference classroom. First, we evaluated the groups formed by 1st grade students and then 2nd grade students. The tests were passed individually during two consecutive days approximately during 30–60 min each session, depending on each student’s performance.

The mathematical proficiency test (Tema-3) was developed only orally, but the students used a pencil and paper to carry out the operations they deemed appropriate to find the result, as stated in the instructions followed to carry out this test. Meanwhile, algorithm tests and problems were presented orally and visually by using several cards. The algorithms were written in numbers, for example_+3 = 5, and the verbal problems were written in Spanish or English depending on the student’s language of instruction, and the numbers that made up the problem were written in figures. In addition, the order of presentation of the tests in both groups was counterbalanced across all participants.

The tests took place subsequently by grades: first grade students were evaluated in April and second grade students in May.

### Results

The analysis of variance was carried out considering two variables: language (not coincident with the language of instruction *group 1* vs. coincident with the language of instruction *group 2*), and grade (first grade vs. second grade). This creates a variable with four possible categories according to group and grade. Once the sample is segmented, we create four groups: first grade when the language of instruction matches their mother tongue and first grade if the language of instruction does not match their mother tongue, and the other two second grades segmented in the same way.

The analysis of the variance between the variable resulting from the tests of Raven and attentional matrices according to the categories indicated above demonstrate that there are differences in mean *F*(3,165) = 10,370, and an MSE = 0.183. with a *p*-value = 0.00, *n*^2^ = 0.15

When comparing the groups by means of the Bonferroni test, we can see how in first and second grades there are no significant differences with a *p*-value in first grade coincident language and first grade not coincident language *p*-value = 1.00, and in second grade coincident language with second grade not coincident language with a *p* > 226. Therefore, the differences can be seen between the first and second grades. For example, first grade not coincident language with second grade not coincident language, and second coincident language have a *p*-value = 0.00 and *p*-value = 0.02, respectively.

The attention test evaluated with the ‘face tests’ showed us again that first grade groups are similar *T*_167_ = 1, 251, and an MSE = 0.424 we find a *p* > 0.213, assuming equal variances.

In second grades, we found similar results *T*_87_ = 0.607 and an MSE = 0.217 we found a *p* > 0.84, assuming equal variances.

We would like to highlight that students in first grade whose mother tongue matches with the language of instruction and the other that do not match the language of instruction are quite similar in the IC level and attentional processes. In the same way, we found similar results between second grade students, when they are segmented in terms of the language of instruction.

The different mathematical tests have been subsequently analyzed. Once the relevant statistics were applied, we obtained three different types of analysis.

In the analysis carried out on mathematical competence × language of instruction, we find two categories in accordance with the grade, given that the items in such a test are different for first and second grade students. We have verified that in first grades there are significant differences *T*_76_ = −4, 44 with a *p*-value *p* = 0.00. However, in second grades *T*_87_ = −0.593 with *p*-value of *p* = 0.554, there would be no significant differences in this competence.

Observing the higher effect of the language of instruction regarding mathematical competence, we carried out the analysis with Cohen’s *D* that in first grades *r* = 0.45, and in second grades *r* = 0.06. Although none of them have a moderate effect, we see a clear decrease in second grade with respect to first grade, as it was the case with problem solving.

Regarding the resolution of algorithms, language of instruction and grade, we found significant differences between the grades. The ANOVA performed showed us the following results *F*(3,165) = 22.56; MSE = 0.634. With a *p*-value = 0.00 and *n*^2^ = 0.29.

**FIGURE 2:**
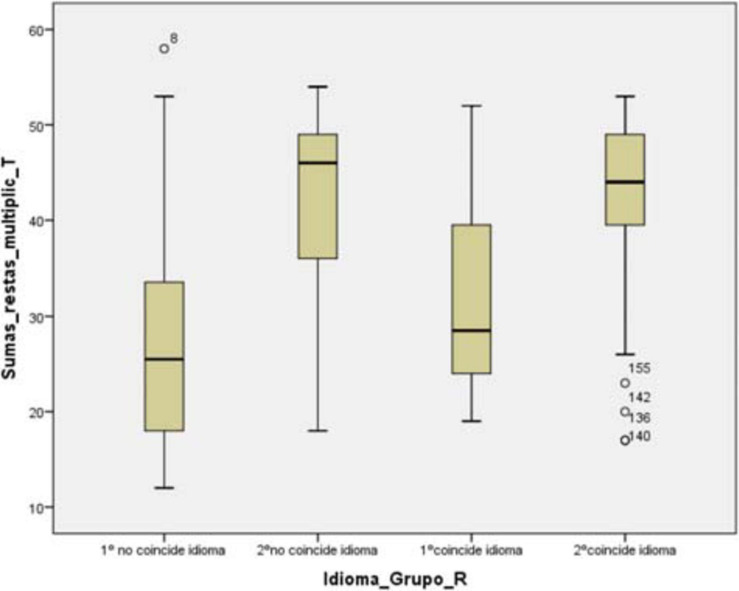
Algorithm resolution × instruction language × grade. Source: Prepared by the authors on the basis of data.

Specifically, according to Bonferroni test, in first grade groups, there are no significant differences with *p*-value = 0.404, and in second grade groups there are no significant differences with a *p*-value = 1.00. Statistically relevant differences occur between first grade groups and second grade groups. There are significant differences with a *p*-value = 0.00 in first grade groups whose language is non-coincident with the second-grade groups non-coincident language, and there are differences with a *p*-value = 0.00 in the first grade group does not match language with the second grade group matches language. Therefore, we found the same *p*-value = 0.00 when first grade groups coincident language is compared with second grade group non-coincident language and second grade group coincident language (Figure 2).

In problem solving, language of instruction, grade we find significant differences in first grade. *F*(3,165) = 8.15 and an MSE = 0.107 with *p* = 0.005 and *n*^2^ = 0.12, therefore, the first grade group whose language of instruction does not match with their mother tongue would differ from the rest, according to the Bonferroni test showing us some *p*-value in first grade *p* < 0.05. Among the other groups there are no indications that there are significant differences.

**FIGURE 3:**
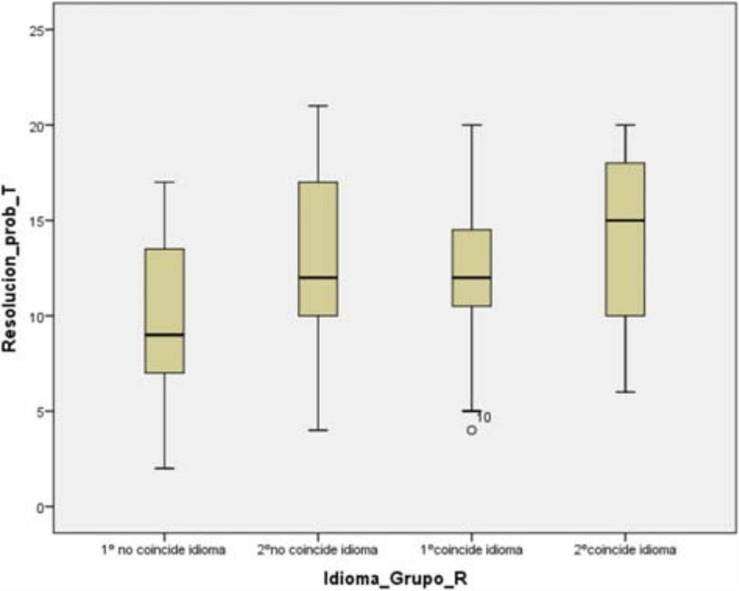
Problem solving × language of instruction × grade. Source: Prepared by the authors on the basis of data.

As a complement to what has been pointed out, we can see in the following graph how the mean of first grade (non-coincident language) clearly differs from the average of the rest of the groups (Figure 3).

Observing the size of the problem-solving effect with respect to the language of instruction, we obtain through the completion of Cohen’s *D* that, in first grade the language of instruction has an effect *R* = 0.32 and in second grade the *R* = 0, 15. Although none of them have a moderate effect, we see a clear decrease in second grade group with respect to first grade group, which is consistent with the results obtained in the previous paragraph, since the effect is trivial.

In the following table we provide a summary of the exploratory statistics referring to problem solving depending on their classification, grade and language of instruction used. We can see that the results at a global level are better when the language of instruction coincides with the mother tongue than when it does not coincide.

Along the study, we also measured the time it took first grade students to carry out the verbal problems. The average time was higher in the group in which the language of instruction did not coincide with their mother tongue. In order to accomplish it, we performed the statistical analyses: instruction, group x time in which we verified that there are significant differences *T*_78_ = 6, 76 with *p* < 0.00. Subsequently, a linear regression is performed in which the time in minutes is on the vertical axis and the resolution of problems on the horizontal axis. As you can see in Table 1.

**TABLE 1:** Descriptive statistics by language coincidence (yes/no) (M, mean; SD, standard deviation).

			***n***	***M***	***SD***	**Mín.**	**Máx.**
**1st grade**							
Type	Change	No language of instruction	44	5.27	1.65	2	8
		Language of instruction	36	6.67	1.15	4	8
	Equalization	No language of instruction	44	1.70	1.27	0	4
		Language of instruction	36	2.06	1.07	0	4
	Combination	No language of instruction	44	0.55	0.76	0	2
		Language of instruction	36	0.86	0.80	0	2
	Comparison	No language of instruction	44	2.14	1.86	0	6
		Language of instruction	36	2.69	1.67	0	6
	Result	No language of instruction	44	4.57	1.39	2	7
		Language of instruction	36	4.83	1.28	2	7
**2nd grade**							
Type	Change	No language of instruction	49	6.24	1.61	3	8
		Language of instruction	40	6.78	1.48	3	8
	Equalization	No language of instruction	49	2.20	1.12	0	4
		Language of instruction	40	2.35	1.23	0	4
	Combination	No language of instruction	49	0.98	0.72	0	2
		Language of instruction	40	1.38	0.77	0	2
	Comparison	No language of instruction	49	3.22	1.62	0	6
		Language of instruction	40	3.50	1.65	0	6
Unknown	Result	No language of instruction	49	5.22	1.48	2	7
		Language of instruction	40	5.48	1.22	2	7

There is a significant relationship between the variables with *p*-value = 0.00. The correlation coefficient between the time variables and the result of the problem solving is *R*^2^ = 0.44. With a positive *R* it shows that there is a direct correlation between time and resolution.

Table 1 shows the coefficients obtained from the multiple linear regression, which simultaneously considers independent variables, resolution according to language, resolution language and resolution problems and as a dependent variable time only language resolution is significant to explain time with a *p*-value = 0.02. Proving 67% of the variance of the model.

Here we can also check problem-solving effects when it coincides with time, being at a lower time when a higher resolution of problems occurs (Table 2).

**TABLE 2:** Summary of the results of the multiple linear regression with all the predictor variables.

	***β_*i*_***	***p***	***R*^2^**	***F***	***df***	***f*^2^**	***1−*β**
**Dependent variable: time**							
Complete model	0.000**	0.44	22.34	3.76	1.05	1.00	
Constant		0.000					
Resolution_ language	–0.745	0.020**					
Language	2.22	0.420**					
Resolution_ Problem_time	0.081	0.571**					

β_*i*_, standardized regression coefficient; R^2^, R fitted square; df, degrees of freedom; f^2^, effect size; 1−β, statistical power. *p < 0.05, **p < 0.01. Source: Prepared by the authors on the basis of data.

## Discussion

The main aim of the present study is to analyze if the language of instruction used in a particular subject-matter, in this case mathematics, can influence the development of concepts, and consequently, the student’s math performance at early ages. The results found in this study are in line with those developed by De Corte and Verscheffel (1991), where the influence of language on mathematical processes is evidenced. However, this research allows us to further clarify these results by selecting samples of different ages (first and second grades in elementary education), and evaluating different mathematical tasks (mathematical competence, operations or algorithms and verbal problems).

We performed the measurement by using the same language of instruction in which mathematics is developed, that is, in the school where math is taught in English the measurement was in this language, and in the school where Spanish is used as the language of instruction, the evaluation was carried out in the same way. Prior to these tasks, each student was provided with a series of instructions on the development of the test in the same language of the measurement, which allowed a previous activation of Spanish or English in each case.

The results show how the influence of the language of instruction is different depending on both the task and the grade. First-graders score lower in mathematical proficiency, algorithm solving, and problem solving when their language of instruction does not coincide with their mother tongue. However, these differences begin to decrease in the following year. In second graders, we see that there are not significant differences in any of the three previous blocks. Nevertheless, it must be pointed out that the performance is still lower than the reference 2nd grade group whose language of instruction coincides with the mother tongue.

In those tasks related to the mathematical competence (i.e., numbering, comparison of quantities, informal calculation, basic concepts, literacy of quantities, strategy of numerical facts, and formal calculation) we find statistically significant differences in first grades in accordance with their language of instruction. The students who show more competent are those taught in the language of instruction coinciding with their mother tongue, but these differences diminish in second grade groups. In this case, the students whose mother tongue coincides with the language of instruction continue to have higher performance than the students of the same grade whose language of instruction does not match; however, in this case these differences are no longer significant.

In the tests given to evaluate arithmetic operations, they had a similar developmental execution. In first grade groups, the resolution of tasks is similar and in the second grade group whose mother tongue does not match with the language of instruction has a slightly higher performance, but there are no significant differences. This indicates that there are no differences in those tasks in which language is not as relevant when it comes to successfully solving the task. Concerning problem-solving tasks, the groups in which their mother tongue matched their language of instruction, solved the tasks more quickly and more efficiently. In first grade groups they obtained significant differences in the mean, however, those differences were reduced in second grade groups.

The results lead us to think that the language of instruction has a direct influence on the development of mathematical thinking, but we see that it is not revealed similarly in all learning. We find tasks such as solving algorithms where the difference between all the groups is quite shorter. One of the reasons we can argue is that students could perform the calculation tasks in their dominant language, regardless of the language in which they were taught. This comes to corroborate what was stated by Van Rinsveld et al. (2015), who affirms that the domain of calculus in the first language seems to follow a continuous development regardless of the language in which formal mathematics is taught. Or else, it could be due to students learning new facts about numbers in one language that retrieve them as efficiently in both languages. It seems that learning responses to logarithms is based on representations that are independent of the language (Spelke and Tsivkin, 2001). While data were being collected, both questions were observed, since there were students who calculated in the dominant language and subsequently translated the result into the English language, and other students, in contrast, executed in the language of instruction in a similar way to those evaluated in the non-coincident language of instruction.

However, problem solving where language has a fundamental role for concept understanding as well as working memory (Swanson et al., 2019), performance is always better when the dominant language is the mother tongue. This reaffirms the idea that children exposed to two languages in which one of them is not a dominant language, will have difficulties in alternating between them when they need it (Costa and Santesteban, 2004). We have to take into account that problem-solving tasks are the most complex since they demand more processes at a cognitive level, a situation that must added to the fact that students must do them in a second language. This reasserts the findings found by Swanson et al. (2019) that suggest how the students who did not master the language, experienced delays in accessing the language concerning the contexts shown in the problems since they had to inhibit the other language. The results show that first grade students whose language of instruction coincides with their mother tongue solve problems faster and are more successful than students with non-coincident language. This result is in line with the results found in Frenck-Mestre and Vaid (1993), Geary et al. (1993), Van Rinsveld et al. (2016) in which the resolution of mathematical tasks was faster and more precise when it is performed in the dominant language. This may indicate that students who have a greater command of the language and understanding of the situation presented, fewer complex procedures were required in their resolution (Van Rinsveld et al., 2016).

We can observe that the previous situation is very frequent and happens independently of the type of problem, and that in all classes of problems students’ performance in the non-dominant language of instruction is lower. However, we can see how the differences vary as the task is becoming simpler. For example, in exchange problems that students find easier than comparison problems, the difference between groups within the same grade is bigger. In contrast, the differences between groups tend to be shortened in comparison problems, since all of them find this task more complex and the error occurs more frequently in all groups. Neither should we forget the learning factor, for this would explain why in first grade groups the differences in problem solving are greater than in second grade groups. The tasks have been solved more effectively by the students in second grade groups than those in first grades, although this result could be expected due to the maturity that students experience during a school year. This has been studied and analyzed in a multitude of investigations from Piaget (1946) to the present-day Bermejo (2018).

Based on Van Rinsveld et al. (2016) students’ performance when solving problems may be due, partly, to the fact that, during the previous school year they had been training in the same type of tasks that allowed them to come up with resolution schemes according to the different structures or else, due to their improved command of the second language committing minor mistakes. Moreover, concerning language, second grade students have better linguistic command, which let conclude that both the language of instruction and being proficient influences the resolution of the tasks, as Van Rinsveld et al. (2015) also explains. We find studies in the same line demonstrating that the greater the linguistic competence, the more the arithmetic performance is promoted (Frenck-Mestre and Vaid, 1993; Geary et al., 1993). Another question that could explain this fact is the one argued by Bialystok (2018) in relation to bilingual models, whose bilingual experience leads to an adaptation of the central executive component of the working memory model, knowing that this component is essential in problem solving, as stated by Swanson et al. (2019), hence the longer the immersion time in these programs, the better the improvement in performing these tasks.

It is relevant to note that when the differences between first and second grades are smaller, it reinforces the results found in various investigations in which it is indicated that some factors such as the age language acquisition, as well as that of the language of instruction seems to be decisive in the use of language when solving mathematical problems in bilingual contexts (Bernardo, 2002; Campbell and Epp, 2004; Salillas and Wicha, 2012; Van Rinsveld et al., 2015).

### Theoretical Implications

From our point of view, the results obtained would have at least two main relevant theoretical applications.

First, our results support the models in which the influence of the instructional language is determined by the type of task that is performed. Although in our results we see that performance is lower on almost all occasions, we can observe that even in the same task such as problem solving that involves the same cognitive processes in the resolution, we can find differences associated with its complexity.

Second, and very importantly, age influences on how information is retrieved according to the language of instruction. The verbal component of Dehaene’s triple code model in the early ages has a greater influence of the dominant language than that of instruction. In the algorithm-solving tasks, the students who were evaluated in the non-dominant language carried out the task in the dominant language to subsequently modify their response in the non-dominant or second language. Therefore, this task was solved more successfully in these groups than others implying an improved linguistic component. Although previous research confirm that a person accesses mathematical concepts more efficiently when they are retrieved in the language of instruction (Dehaene et al., 1999; Spelke and Tsivkin, 2001), in this case it is different depending on the language used, that is if it is the dominant language or not, as it has been found in the results obtained. Some of these studies have been carried out in adult population, which would indicate that at an early age the dominant language may be much more influencing than the language of instruction. However, we see that these differences draw closer in higher grades, which may indicate a tendency to minimize the effects as language proficiency increases compared to mathematical learning.

Finally, we can remark that another implication pulled out from the present study is that the differences in mathematical performance are shortened as time progresses when the language of instruction is different from the mother tongue. This reinforces Bialystok (2018) suggesting that the bilingual experience leads to an adaptation of the central executive component of the working memory model; therefore, some tasks such as problem solving improve as there is greater adaptation.

### Practical Implications

We believe that learning mathematics in bilingual environments is a far more challenging than in monolingual classrooms, but perhaps, we must accept certain aspects related to the child’s language development that will let them reach a learning stage where the language of instruction comes in and becomes more influential than the individual’s dominant language.

One of the practical implications of our results lets us establish the appropriate differences in the mathematical tasks with a lower performance of the students in programs in which the language of instruction is not the dominant one, since in this way we could detect the learning difficulties associated with mathematical concepts as well as those associated with second language acquisition. This could set the course for educational interventions since, depending on the differential diagnosis in both areas, it will allow us to carry out more individualized programs.

We think that it would be important to keep these aspects in mind when developing bilingual programs for the teaching of mathematics, and we completely agree with the criticism given to the bilingual programs by Van Rinsveld et al. (2015), since many language immersion programs assume that the contents are linguistically independent to be transferred in what they call “*Learners’ mental language*.”

Accordingly, any subject could be taught in an additional language without having any effect on the development of the concepts, and as we have presented in the results of this research, we should take into account certain implications. From our point of view, daily life mathematical problems in which the influence of language is very high, could be worked in workshops, in order to reinforce transfers between the different linguistic structures that define the mathematical problems and the representations that are deduced from them until the student fully understands the problems worked.

Nevertheless, we also find highly relevant that students do not carry them out with the algorithmic form since they have an abstract visual representation that can be the link between both representations. In addition to this, as Van Rinsveld et al. (2015) suggests, the recovery of arithmetic facts can be independent of verbal codes or as sufficiently automated in both languages so as to have a similar competence in either of them. As Cerezci (2020) exposes, improving the quality of early-year instruction in mathematics will allow us not only to improve student’s results but also fill in the existing gap in the theoretical approach. In this way, it will permit to identify and document the main characteristics of quality instruction in teaching mathematics to early year students within bilingual programs.

### Limitations

In the present study there are at least two limitations that need to be mentioned. On the one hand, we mean that the design used in this research is transversal rather than longitudinal. We think that in order to study the development of language skills and how they influence the performance of certain subjects, such as mathematics, it is more enriching to carry out longitudinal studies that analyze the complicated changes that may occur in schoolchildren over a few years. However, we are all aware of the advantages and disadvantages of both designs. And on the other, the study has had a limitation on the linguistic data collection, as there are no data on the students’ linguistic competence which could let us establish correlations on their mathematical competence, whether or not their language of instruction coincides.

## General Considerations

Previous research has shown diverse results on the influence of language on the development of mathematical thinking in bilingual people, but virtually none of them has studied it at an early age when the development of language, number and arithmetic occur simultaneously and during the same period of time.

In the resolution of algorithms, it has been possible to verify how the students who were taught mathematics in a second language had a similar resolution to those that the teaching language coincided with their mother tongue, but it was not the case in the resolution of verbal problems or in the tests of mathematical competence. We could further say that, in both verbal problems and mathematical competence tests, the differences between the two groups were shorter in higher grades. The time of language acquisition, the language of instruction and the individual’s competence affects the resolution of mathematical problems (Salillas and Wicha, 2012; Van Rinsveld et al., 2015). This fact should be taken into account when implementing bilingual programs in schools to focus on looking for ways to minimize the risks of learning the subjects and face the subsequent evident benefit.

This study would open several lines of research. On one hand, it would open a line to conduct longitudinal studies allowing us to know in depth the learning of mathematics in a second language and the resulted implications in all academic years. On the other hand, it could also lead to study whether actions such as the reformulation and simplification of the structure of daily life verbal problems can improve their resolution in a second language in the same way as it is performed in the mother tongue. Finally, from the educational point of view, complementary programs could be developed to allow students to overcome mathematic learning limitations when solving daily life problems within bilingual programs.

## Data Availability Statement

The datasets generated for this study are available on request to the corresponding author.

## Ethics Statement

Ethical review and approval was not required for the study on human participants in accordance with the local legislation and institutional requirements. Written informed consent to participate in this study was provided by the participants’ legal guardian/next of kin.

## Author Contributions

VB, PE, and IM contributed to give life, conception and shape to the study. PE and IM conducted the study and collected the data. PE organized the database and performed the statistical analysis. PE wrote the first draft. IM complemented the second draft with further theoretical support. IM wrote the English version. VB, PE, and IM wrote sections, read and approved the submitted section. All authors contributed to the article and approved the submitted version.

## Conflict of Interest

The authors declare that the research was conducted in the absence of any commercial or financial relationships that could be construed as a potential conflict of interest.
